# The Effects of an Online Mind-Body Training Program on Stress, Coping Strategies, Emotional Intelligence, Resilience and Psychological State

**DOI:** 10.1371/journal.pone.0159841

**Published:** 2016-08-01

**Authors:** Ye-Ha Jung, Tae Min Ha, Chang Young Oh, UI Soon Lee, Joon Hwan Jang, Jungwon Kim, Jae-Oh Park, Do-Hyung Kang

**Affiliations:** 1 Department of Neuropsychiatry, Seoul National University Hospital, Seoul, Republic of Korea; 2 Department of Psychiatry, Seoul National University College of Medicine, Seoul, Republic of Korea; 3 Department of Brain Education, Global Cyber University, Cheonan, Republic of Korea; 4 Department of Occupational and Environmental Medicine, Kosin University College of Medicine, Busan, Republic of Korea; 5 Department of Occupational Health Research, Occupational Safety and Health Research Institute, Ulsan, Republic of Korea; Defence Science and Technology Group, AUSTRALIA

## Abstract

The goal of this study was to evaluate the effects of an online mind-body training (MBT) program on participants’ stress, anger, coping strategies, emotional intelligence, resilience, and positive and negative affect. Forty-two healthy women participated in an online MBT program for approximately 8–10 minutes a day for 8 weeks; a control group of 45 healthy women did not participate in the program. Self-report psychological questionnaires were administered before the beginning of the program and at 4 and 8 weeks following its onset. Data from the MBT group and the control group were compared using repeated measures ANOVA and Student’s t-tests. Significant time x group interaction effects were found with respect to stress, coping strategies, anger, emotional intelligence, negative affect and resilience. These results demonstrate beneficial effects of the online MBT program and significant improvements in the psychological capabilities of participants compared with the control group. The effects of online MBT program were similar with those of the previous offline MBT in psychological aspects, suggesting further studies for neuroscientific evidence related stress and emotion of online MBT effects.

## Introduction

A variety of mind–body training (MBT) programs have been reported to decrease perceived stress and improve mood [1]. MBT programs, which may involve mindfulness based on mind–body skills or yoga-based lifestyle intervention, have been shown to reduce stress [2] and anxiety [3]. In addition, mindfulness-based stress reduction and healing arts programs involving cancer outpatients have shown significant effects with regard to anxiety, anger, overall stress symptoms, and mood disturbances [4]. Furthermore, an integrative coping and resiliency program demonstrated simultaneous effects of stress reduction and improved coping and mindfulness [5–6].

The Internet offers many advantages as a medium for easy participation in MBT and is not subject to the limitations of time and space that are inherent to traditional group training. There is evidence of the impact of online training in mind–body skills in terms of significant improvements related to stress, mindfulness, and participants’ confidence in their ability to provide calm and compassionate care, with emphasis on the cost-effectiveness and convenience of online programs [7]. The same researchers have also reported effects of online mind–body skills training on resilience, mindfulness, and empathy [8].

Many workers who are required to perform emotional labor suffer from emotional burnout and occupational stress. Emotional labor is a job that employees display required emotions toward customers or others [9]. Emotional labor is positively correlated with both daily stress and 'interaction stress' levels [10]. Occupational stress and emotional labor are both related to mental health [11]. Occupational stress stemming from the psychosocial work environment is stress related to one's job and is associated with anxiety [12] and depressive symptoms [13].

Stress symptoms appear to be related to ineffective coping and emotional intelligence [14]. Emotional intelligence is the capability of individuals to recognize their own and others’ emotions, to discriminate between different feelings and label them appropriately, and to use emotional information to guide thinking and behavior [15]. Low emotional intelligence is associated with worry states and avoidance coping strategies [16] and greater degrees of psychological stress [17]. It has been suggested that emotional intelligence acts as a moderator between stress and psychological health [18]. Increased emotional competence enhances effective coping strategies for dealing with stress [19], and problem-focused coping has a positive effect with regard to alleviating psychological distress [20]. In addition, resilience, which is defined as an individual's ability to properly adapt to stress and adversity, may be viewed as a measure of stress coping ability and may be an important target of treatment for anxiety, depression, and stress reactivity [21].

The current online MBT program was designed to incorporate MBT content and skills that have been shown to have beneficial effects related to stress, positive affect [22, 23], and the structure and function of the brain [24, 25]. As MBT can enhance the state of restful alertness that facilitates a high degree of awareness of the body, breathing, and external instructions [26], this MBT program was designed to incorporate physical and emotional relaxation exercises, physical movements, deep breathing exercises, and meditation practices suitable for a healthy population. There were three theoretical aspects that underpinned the MBT protocol. The first section consisted of body movements designed to enhance the health of the brain stem, which is connected to the autonomic nervous system. It was designed to ameliorate the physical symptoms resulting from the secretion of stress hormones and to improve the condition of the body [27–28]. The second section consisted of breathing exercises designed to facilitate the health of the limbic system which can become disrupted under the influence of stress [29–30]. To this end, the exercises release negative emotions and enhance positive ones. The third section consisted of meditation practices designed to enhance the health of the cerebral cortex, which is associated with memory distortions related to stress and emotional balance [31–32]. It mitigates the negativity of thoughts derived from stress to improve the regulation of emotions and the ability to handle stress.

**Fig 1:**
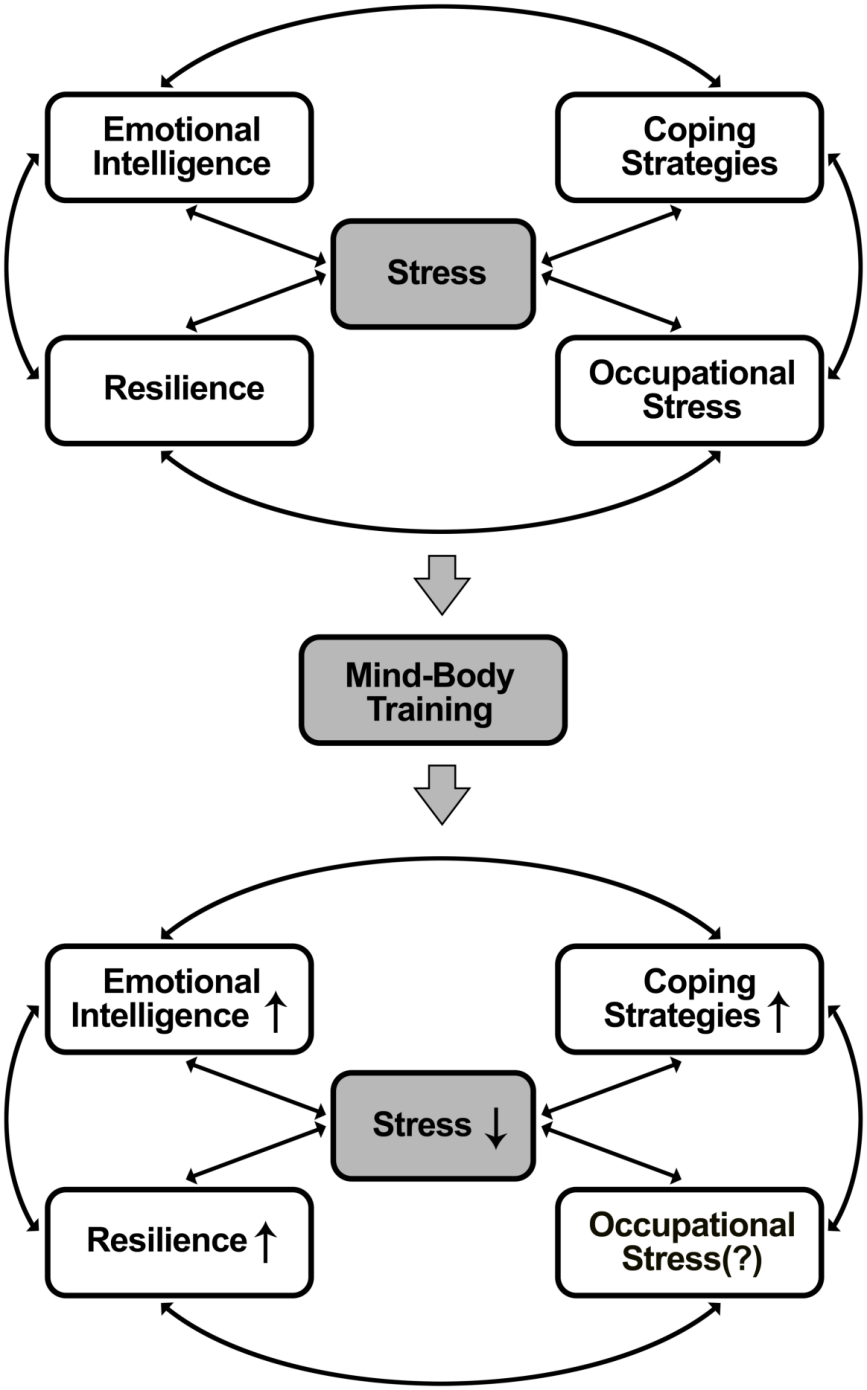
Proposed model of the effects of MBT on perceived stress, coping strategies, emotional intelligence, and resilience.

Subjects in the MBT group participated in the online program for 10 minutes a day, 5 days per week, over a period of 8 weeks. The 8-week duration of the program was in line with most other programs, but our program implemented the novel feature of using 10-minute daily online videos. Although the impact of other online training programs has been studied [7–8], it has yet to be determined whether this online MBT program, which was created based on our previous offline MBT program, is as effective as the offline group training [22–25]. We hypothesized that this online MBT would have beneficial effects involving stress reduction, increased use of adaptive coping strategies, increased emotional intelligence, and enhanced resilience (Fig 1). Moreover, the impact of implementing online training in a time-compressed fashion, based on 10 minutes a day, is unknown. The aim of this study was to investigate whether our current online MBT program is indeed beneficial with regard to its effects on stress, anger, coping strategies, emotional intelligence, resilience, and positive or negative affect. We assessed the effects of the program on participants after 4 weeks (short-term effects) and 8 weeks (longer-term effects).

## Methods

### Subjects and study design

All participants in the control and MBT groups participated voluntarily in this research study. The MBT group consisted of 42 subjects (all women) who consistently took part in the 8-week online MBT program. The control group consisted of 45 subjects (all women) who did not participate in the program or receive any other intervention during the 8-week period. All of the participants in both groups worked in the hospital; most of them were employed as nurses or in jobs associated with caring for, or supporting, patients or members of the hospital—that is, in jobs that required emotional labor. Exclusion criteria included a history of psychosis, head trauma or neurological disorders. Psychiatric disorders among the participants were assessed using the Symptom Checklist-90-Revised (SCL-90-R) [33] and participants who scored higher than 70 (in T-scores) were excluded from this study. Subjects who were currently participating or who had participated in any kind of MBT in the previous 3 months were excluded. Within the MBT group, participants who failed to practice the MBT program as required on more than 4 occasions during either the first or second 4-week segment of the program were dropped from the study. This study was approved by the Institutional Review Board at Seoul National University Hospital, and written informed consent was obtained from all participants after the procedures had been fully explained. This study was conducted in accordance with the Declaration of Helsinki. The experimental and control groups were recruited via notices posted on the bulletin board of the hospital. The advertisement recruited volunteers for participation in an experimental mind–body healing and training program or for completing psychological tests without participating in the program (control group). Each group was recruited separately. This study did not use a randomized controlled trial design. Both groups participated in each of the three self-reported psychological tests administered at baseline, at 4 weeks and at 8 weeks. The tests were individually administered offline in a paper-and-pencil format.

### The content and methods of the MBT program

Those in the experimental group participated individually in an online MBT program at home or at their workplace for 8 weeks. The program was performed once per day, 5 days per week, with each daily session lasting 10 minutes. Participants were taught the techniques by watching MBT experts on an online video and then following the MBT protocol for each 10-minute program. Participants in the MBT program were provided with a checklist on which to record their daily practice, which they submitted after the 8-week training. The program was based on mind–body training, a kind of movement-based meditation designed to facilitate the relaxation of the mind and the release of negative emotions through natural rhythmic movements and a focus on bodily sensations [22]. The program consists of 10 phases: brain relaxation exercise 1, brain relaxation exercise 2, brain rejuvenation exercise, relaxation breathing, chest breathing, meditation with self-watching, energy-focused meditation, brain-refreshing meditation, meditation for balanced brainwaves, and meditation for emotional release. The first phase, brain relaxation exercise 1, was designed to enhance flexibility and blood circulation through stretching the muscles of the body. It consists of postures and motions that help relax the neck, shoulders, and lower back which can easily become tight when workers are exposed to conditions associated with burnout. The second phase, brain relaxation exercise 2, was also designed to enhance flexibility and blood circulation through stretching the muscles of the body. Additionally, this exercise includes movements that pull the tips of the toes toward the body and then push them outward, and bringing the hands to the armpits and dropping them down while relaxing the tension in the shoulders. The third phase, the brain rejuvenation exercise, rejuvenates the relaxed muscles and increases the energy level of the body. It consists of exercises such as body tapping, toe-tip tapping, and clapping hands. In the fourth phase, relaxation breathing is used to deepen and slow the cycle of breathing which becomes short and shallow due to stress. It consists of taking one breath in six segments and breathing out quickly. Both this breathing exercise and its reverse are repeated 3–4 times. In the fifth phase, chest breathing is performed to reduce emotional stress by integrating breathing and consciousness. When breathing in, focus should be on the chest; when breathing out, focus should be on the abdomen. As this exercise is repeated 4–5 times, breathing can become more comfortable. It is easier to focus on and follow this exercise if the right hand is placed on the chest and the left hand is placed on the lower abdomen. The sixth phase, meditation with self–watching, is performed to relax muscular tension by focusing on the body. Participants focus on each part of the body to increase relaxation as the instructor names it, moving from the top of the head to the tips of the toes. This exercise is repeated 2–3 times. The seventh phase, energy-focused meditation, involves feeling the energy of the body and establishing peace of mind. It consists of the motions needed to move the hands 5 cm above the chest, move them apart, and move them closer while feeling the sensation of one’s palms. The eighth phase, brain-refreshing meditation, is employed to clear the stress-related fogginess of the brain. As participants assume a meditation posture, moving the hands apart and closer, they imagine and say the following: "Pure breath is coming into my head, my brain is expanding." They then breathe out with a "Whoo" sound and imagine, "Foggy gas is coming out from my brain through my mouth." It is important that a positive brain state is imagined. The ninth phase, meditation for balanced brainwaves, is aimed at converting the beta waves associated with stress to the alpha waves associated with relaxation and concentration. The first step involves breathing in, holding the breath, and relaxing the neck as the breath is released. The second step involves moving or shaking the head from side to side in a comfortable and relaxing manner. The third step consists of quietly stopping all motion and relaxing the mind. The tenth phase, meditation for emotional release, was designed to erase and reduce the influence of the images of stressful events or people stored in the brain. First, participants recall the image of a negative experience and notice its position and size. They use their imagination to make it as small as a pea and blow it away while saying, "Whoo." This is repeated several times. The experimental subjects repetitively practiced each of the 10 phases four times over the course of 8 weeks.

### Psychological measures

#### Korean Occupational Stress Scale (KOSS)

The KOSS was used to evaluate the level of occupational stress present in the psychosocial work environment [34]. The KOSS includes forty-three items (score range: 0–100) scored using conventional 1-2-3-4 Likert scores and the following eight subscales developed through factor analysis and a validation process: physical environment (three items), job demand (eight items), insufficient job control (five items), job insecurity (six items), interpersonal conflict (four items), organizational system (seven items), lack of reward (six items), and occupational climate (four items). Higher scores indicate higher levels of occupational stress. Internal consistency alpha scores ranged from 0.51 to 0.82 in a previous study [34]. The Cronbach's alpha in the current study was 0.80 for the full scale. The Cronbach's alpha for the eight subscales ranged between 0.27 and 0.82: physical environment (0.40), job demand (0.84), insufficient job control (0.27), job insecurity (0.51), interpersonal conflict (0.65), organizational system (0.82), lack of reward (0.71), and occupational climate (0.68).

#### Stress Response Inventory (SRI)

Participants’ psychological response to stress was measured using the SRI [35]. The inventory consists of 39 items (score range: 0–156) categorized into 7 factors: fatigue, tension, frustration, anger, depression, somatization, and aggression. Higher scores indicate higher levels of perceived stress. The Cronbach's alpha for the seven subscales ranged between 0.76–0.91 and 0.97 for the total score in a previous study [35]. The Cronbach's alpha in the current study was 0.96 for the full scale. The Cronbach's alpha for the seven subscales ranged between 0.72 and 0.89: fatigue (0.72), tension (0.73), frustration (0.89), anger (0.77), depression (0.83), somatization (0.75), and aggression (0.77).

#### Coping Strategy Indicator (CSI)

The Korean version of the CSI [36] was used to measure coping strategies. The CSI addresses a broad range of problem- and emotion-focused coping strategies that people might use in dealing with stressful situations. The inventory consists of 33 items rated on a three-point self-administered scale: not at all (1), a little (2), and a lot (3). This instrument was designed to assess three basic modes of coping: problem solving (11 items, score range: 11–33), social support (11 items, score range: 11–33) and avoidance (11 items, score range: 11–33). Problem-solving coping is problem focused, avoidance coping is emotion focused and social-support coping can either be problem or emotion focused. The Cronbach’s alpha coefficients indicated adequate internal consistency for each of the subscales, with values ranging from 0.86 to 0.98 for problem solving, from 0.89 to 0.98 for seeking social support, and from 0.77 to 0.96 for avoidance in a previous study [36]. The Cronbach's alpha in the current study for the three subscales ranged between 0.63 and 0.93: problem solving (0.93), social support (0.92) and avoidance (0.63).

#### State–Trait Anger Expression Inventory (STAXI)

The Korean version of STAXI was used to determine latent classes of anger symptoms [37], based on 10-item scales to measure the intensity of anger as an emotional state (state anger: 10 items, score range: 10–40) and individual differences in anger proneness as a personality trait (trait anger: 10 items, score range: 10–40). The state anger scale was designed to measure a psychobiological state consisting of subjective feelings varying in intensity from mild irritation or annoyance to intense fury and rage. The trait anger scale measures individual differences in how often state anger is generally experienced. Each item is rated on a four-point Likert-type scale, with higher scores indicating higher levels of state or trait anger. The Cronbach's alpha coefficient for internal consistency was 0.84 in a previous study [38]. The Cronbach's alpha in the current study was 0.84.

#### Emotional Intelligence Questionnaire (EIQ)

Emotional intelligence was measured using the Korean version of the instrument developed [39] based on the ability model of emotional intelligence [40]. Emotional intelligence was assessed by means of 50 questions. Each question was scored on a five-point Likert-type scale, with higher scores indicating greater emotional intelligence (score range: 50–250). The scale was categorized into the following five factors, each of which consisted of 10 questions: emotional perception and expression, empathy, emotional thinking, emotional application, and emotion regulation. Split-half reliability coefficients ranged from r = 0.80 to 0.91; the value for the entire measure was r = 0.91 in a previous study [41]. The Cronbach's alpha in the current study was 0.89 for the full scale. The Cronbach's alpha for the five subscales ranged between 0.61 and 0.79: emotional perception and expression (0.63), empathy (0.61), emotional thinking (0.77), emotional application (0.79), and emotion regulation (0.75).

#### Positive Affect and Negative Affect Schedule (PANAS)

The Korean version of PANAS [42] was used to measure positive and negative affect. This scale consists of 20 items describing various feelings and emotions in terms of 10 positive (interested, alert, attentive, excited, enthusiastic, inspired, proud, determined, strong, active) and 10 negative affective descriptors (distressed, upset, guilty, ashamed, hostile, irritable, nervous, jittery, scared, afraid). Each question was scored on a 5-point Likert-type scale, with higher scores indicating greater degrees of positive (score range: 10–50) or negative affect (score range: 10–50). Cronbach’s alpha reliabilities were all acceptable, ranging from 0.86 to 0.90 for PA and from 0.84 to 0.87 for NA in a previous study [42]. The Cronbach's alpha in the current study was 0.82 for PA and 0.81 for NA.

#### Connor–Davidson Resilience Scale (CDRS)

The Korean version of CDRS [21] comprises 25 items (score range: 25–125), each rated on a 5-point scale (range: 1–5). The resilience scale is categorized into five simplified factors. Factor 1 (tenacity for high standards) reflects the notion of personal competence, high standards, and tenacity. Factor 2 (strength for overcoming stress) corresponds to trust in one’s instincts, tolerance of negative affect, and the strengthening effects of stress. Factor 3 (positive acceptance) relates to positive acceptance of change and the degree of security in relationships. Factor 4 is related to control and Factor 5 to spiritual influences. The Cronbach’s alpha for the full scale was 0.89 in a previous study [21]. The Cronbach's alpha in the current study was 0.92 for the full scale. The Cronbach's alpha for the five subscales ranged between 0.19 and 0.83: tenacity for high standards (0.83), strength for overcoming stress (0.80), positive acceptance (0.73), control (0.62), spiritual influences (0.19).

### Statistical analysis

The data were analyzed using repeated-measures ANOVA (rmANOVA) (factors: group and time). Tests of within-subjects interaction effects (time x group) were used to identify the longitudinal effects of MBT on various psychological states. Subsequently, tests of within-subjects contrasts (time x group) were carried out to identify effects between level 1 (baseline) and level 2 (4 weeks after program onset), between level 1 and level 3 (8 weeks after program onset), and between level 2 and level 3. Differences between the MBT and control groups as reflected by changes in occupational stress scores over the 8-week period were analyzed using Student’s t-test. Pearson’s correlation coefficient was used to analyze the relationship between two variables. All p-values of less than 0.05 were considered statistically significant.

## Results

No significant age differences were observed between the MBT (mean ± SD: 35.0 ± 8.20) and control (mean ± SD: 36.6 ± 7.6) groups. There were no significant baseline differences between the two groups with regard to stress, emotional intelligence, resilience, anger, positive or negative affect, total occupational stress and coping strategies such as problem-solving and avoidance. The MBT group showed significantly lower social-support coping than the control group at baseline (p = 0.023).

To investigate whether occupational stress in the work environment remained similar over the duration of the study, we analyzed variations in occupational stress at 4 weeks and 8 weeks, using a Student’s t-test to compare scores between the MBT and control groups at these times. Total occupational stress did not exhibit any significant between-group differences at either 4 or 8 weeks (Table 1). The factor of interpersonal conflict in occupational stress exhibited a significant difference between the MBT and control groups (Table 1, p = 0.040) only at the 4-week mark, not at 8 weeks. The remaining factors of occupational stress did not exhibit any significant differences between the two groups at either 4 or 8 weeks (Table 1).

**Table 1:** Differences in KOSS scale scores for the MBT and control groups at baseline, 4 weeks and 8 weeks.

	MBT group(M±SD)			Control group(M±SD)			Group difference in mean change from two levels (95% CI)	p
	Baseline	4-week	8-week	Baseline	4-week	8-week		
Physical Environment	44.44±17.70	45.24±15.98	45.77±15.56	44.20±15.98	46.67±15.46	46.42±15.40	4 week: -1.68(-6.67,3.32)	0.507
							8 week: -0.90(-6.43,4.63)	0.747
Job Demand	52.88±20.93	55.75±16.44	55.75±16.02	54.81±12.40	56.67±13.97	56.11±14.20	4 week: 1.03(-3.22,5.27)	0.632
							8 week: 1.58(-3.19,6.35)	0.512
Insufficient Job Control	55.40±12.96	55.56±10.69	54.76±11.92	57.93±2.13	56.44±11.06	54.81±9.84	4 week: 1.64(-3.00,6.29)	0.485
							8 week: 2.48(-2.49,7.44)	0.324
Job Insecurity	50.00±14.04	49.21±14.96	47.09±13.40	53.46±14.71	53.33±13.21	51.98±14.99	4 week: -0.67(-6.53,5.19)	0.821
							8 week: -1.43(-7.33,4.47)	0.632
Interpersonal Conflict	42.46±16.55	38.29±14.14	41.47±12.41	32.96±11.37	34.63±11.78	32.96±8.70	4 week: -5.83(-11.39,-0.28)	**0.040**
							8 week: -0.99(-6.72, 4.73)	0.731
Organizational System	64.51±16.37	59.75±15.25	62.24±14.40	57.67±12.73	55.56±9.30	54.60±10.82	4 week: -2.65(-7.92, 2.63)	0.321
							8 week: 0.80(-4.72, 6.33)	0.774
Lack of Reward	56.48±15.99	52.78±10.71	53.70±11.22	51.98±12.66	52.72±10.37	51.85±12.76	4 week: -4.44(-9.08, 0.19)	0.060
							8 week: -2.65(-8.11, 2.81)	0.336
Occupational Climate	46.63±16.47	45.44±15.20	48.41±17.19	36.11±17.59	38.52±14.47	39.44±13.58	4 week: -3.60(-10.17,2.97)	0.279
							8 week: -1.55(-8.25,5.15)	0.647
Occupational Stress(total)	51.60±7.76	50.25±6.87	51.15±7.57	48.64±7.25	49.32±7.91	48.52±7.64	4 week: -2.03(-4.24,0.19)	0.073
							8 week: -0.33(-3.08,2.42)	0.810

p: p-value, KOSS: Korean Occupational Stress Scale, CI: Confidence Interval

### Effects of MBT on stress, coping strategies, emotional intelligence, and resilience

**Fig 2:**
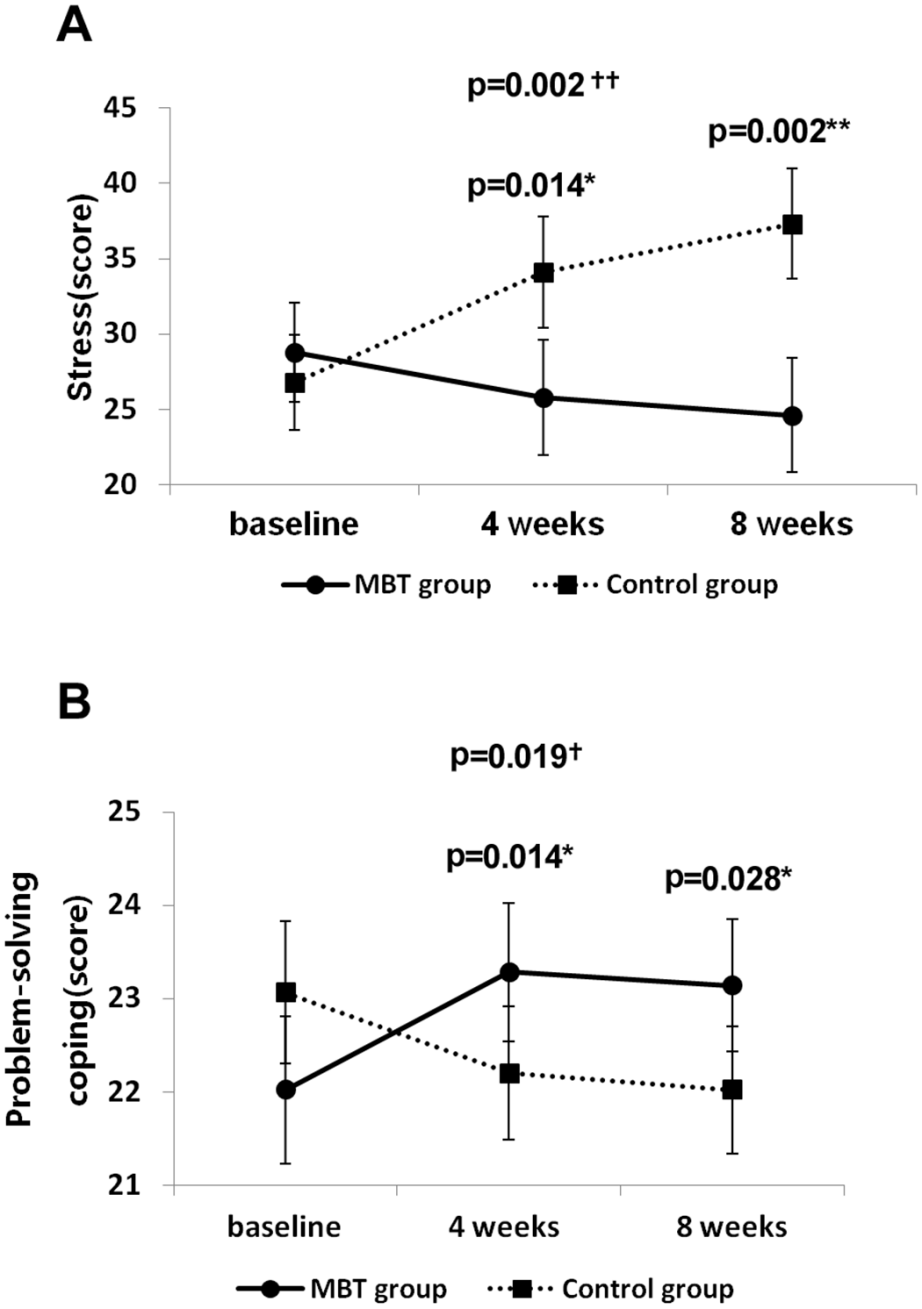
Change in total stress (A) and problem-solving coping (B) scores for the MBT and control groups across the 8 weeks. (A) Significant changes for 8 weeks in stress (total score of 7 factors). (B) Significant changes for 8 weeks in problem-solving coping. †: Tests of within-subjects effects (time x group). *: Tests of within-subjects of contrasts (time x group).

Next, the interaction effects (time x group) were assessed using rmANOVA, followed by tests of within-subjects contrasts comparing baseline vs. 4 weeks, baseline vs. 8 weeks, and 4 weeks vs. 8 weeks. A significant time x group interaction effect (p = 0.002) in total stress level was found, with the contrast test yielding a significant time x group interaction (Fig 2A) when comparing baseline vs. 4 weeks (p = 0.014) and baseline vs. 8 weeks (p = 0.002). Tension, frustration, anger, depression, somatization of stress factors excluding fatigue and aggression showed significant time x group interaction effects (Table 2), demonstrating differences in changes over time between the two groups. Contrast tests using rmANOVA showed a significant time x group interaction with regard to levels of frustration and anger when comparing baseline vs. 8 weeks, but not when comparing baseline vs. 4 weeks, implying that sufficient training time (between 4 and 8 weeks) was needed to observe a reduction in these symptoms (Table 2). Contrast tests using rmANOVA further showed significant time x group interactions with regard to tension, depression, and somatization of stress factors when comparing baseline vs. 4 weeks and baseline vs. 8 weeks (Table 2). During the 8-week experimental period, significant changes were observed in total stress levels (Fig 2A) and most of the factors related to stress (Table 2). A significant time x group interaction was found with regard to problem-solving coping (Fig 2B, p = 0.019), with contrast tests yielding significant interaction effects when comparing baseline vs. 4 weeks (p = 0.014) and baseline vs. 8 weeks (p = 0.028), demonstrating the enhancement of coping strategies among the MBT participants compared with the control group. In addition, a significant time x group interaction was found in social-support coping (Table 2, p = 0.003) indicating that over the course of the 8-week program, significant improvements in coping strategies were observed among MBT participants compared with the control group (Fig 2B, Table 2).

**Table 2:** Differences in SRI, CSI, EIQ and CDRS scale scores for the MBT and control groups at all time points.

	MBT group(M±SD)			Control group(M±SD)			rmANOVA		
	Baseline	4-week	8-week	Baseline	4-week	8-week	Time x Group (p)	Group difference in mean change from two levels (95% CI)	p
SRI									
Fatigue	4.69±3.52	4.86±3.45	4.98±3.67	4.87±2.83	6.13±3.36	6.20±3.45	0.141	Level 1–2: -1.10(-2.39,0.19)	0.093
								Level 1–3: -1.05(-2.39,0.29)	0.124
Tension	4.38±3.81	4.38±4.17	3.90±4.31	3.84±2.85	5.49±3.65	5.87±4.05	**0.007**	Level 1–2: -1.64(-3.06,-0.23)	**0.023**
								Level 1–3: -2.50(-4.27,-0.73)	**0.006**
Frustration	6.10±5.95	5.40±5.36	4.79±4.51	6.04±4.70	7.22±6.40	7.62±5.86	**0.026**	Level 1–2: -1.87(-4.11,0.37)	0.101
								Level 1–3: -2.89(-5.15,-0.62)	**0.013**
Anger	4.14±3.83	3.88±4.91	3.62±3.77	4.18±3.57	5.18±4.18	5.82±4.57	**0.013**	Level 1–2: -1.26(-2.66,0.14)	0.077
								Level 1–3: -2.17(-3.76,-0.58)	**0.008**
Depression	6.52±5.19	5.14±5.60	5.02±5.21	5.04±4.10	6.40±5.39	7.29±5.67	**0.001**	Level 1–2: -2.74(-4.76,-0.72)	**0.009**
								Level 1–3: -3.74(-5.79,-1.70)	**0.000**
Somatization	1.90±2.21	1.52±2.42	1.50±2.09	1.64±2.07	2.33±2.56	2.87±2.74	**0.002**	Level 1–2: -1.07(-1.98,-0.16)	**0.022**
								Level 1–3: -1.63(-2.56,-0.70)	**0.001**
Aggression	1.05±2.21	0.60±2.06	0.81±1.86	1.18±1.90	1.33±1.87	1.64±2.19	0.123	Level 1–2: -0.61(-1.31,0.10)	0.090
								Level 1–3: -0.70(-1.55,0.14)	0.102
SRI (total)	28.79±23.14	25.79±24.58	24.62±22.51	26.80±19.31	34.09±24.80	37.31±26.25	**0.002**	Level 1–2: -10.29(-18.43,-2.15)	**0.014**
								Level 1–3: -14.68(-23.87,-5.48)	**0.002**
CSI									
Problem-Solving Coping	22.02±4.78	23.29±4.98	23.14±4.84	23.07±5.40	22.20±4.63	22.02±4.31	**0.019**	Level 1–2: 2.13(0.44,3.82)	**0.014**
								Level 1–3: 2.16(0.25,4.08)	**0.028**
Social Support	22.48±5.14	23.81±5.24	23.26±4.62	24.98±4.97	23.71±4.43	23.16±3.55	**0.003**	Level 1–2: 2.60(1.01,4.19)	**0.002**
								Level 1–3: 2.61(0.72,4.49)	**0.007**
Avoidance	16.81±2.88	17.26±3.22	16.90±3.27	16.27±3.39	16.87±2.81	17.00±3.13	0.534	Level 1–2: -0.15(-1.29,0.99)	0.798
								Level 1–3: -0.64(-1.88,0.60)	0.309
EIQ									
Emotional Perception and Expression	36.00±4.02	35.36±4.39	36.26±4.66	35.33±4.55	34.29±4.37	34.24±3.97	0.132	Level 1–2: 0.40(-0.91,1.71)	0.543
								Level 1–3: 1.35(-0.08,2.78)	0.064
Empathy	35.43±4.27	35.43±4.99	34.93±4.91	34.93±4.48	34.64±4.08	34.20±4.20	0.928	Level 1–2: 0.29(-1.17,1.75)	0.695
								Level 1–3: 0.23(-1.38,1.85)	0.774
Emotional Thinking	31.62±4.38	33.36±4.20	33.21±4.60	33.20±4.88	33.04±4.16	32.78±4.31	**0.036**	Level 1–2: 1.89(0.31,3.48)	**0.020**
								Level 1–3: 2.02(0.04,3.99)	**0.046**
Emotional Application	33.17±5.63	33.55±5.54	34.10±5.11	32.58±5.46	32.78±4.70	32.67±4.69	0.513	Level 1–2: 0.18(-1.42,1.78)	0.823
								Level 1–3: 0.84(-0.65,2.32)	0.264
Emotion Regulation	32.43±4.87	33.69±5.32	33.83±5.00	34.07±4.97	33.69±3.76	33.49±4.80	**0.033**	Level 1–2: 1.64(0.10,3.18)	**0.038**
								Level 1–3: 1.98(0.22,3.74)	**0.028**
EIQ (total)	168.64±17.58	171.38±20.17	172.33±20.05	170.11±17.52	168.44±16.50	167.38±18.33	**0.039**	Level 1–2: 4.40(-0.31,9.12)	0.067
								Level 1–3: 6.42(0.93,11.92)	**0.022**
CDRS									
Tenacity for High Standards	25.98±4.79	26.76±5.33	27.43±4.81	25.33±4.08	25.18±4.81	24.60±3.99	**0.012**	Level 1–2: 0.94(-0.54,2.43)	0.211
								Level 1–3: 2.19(0.66,3.71)	**0.005**
Strength for Overcoming Stress	20.21±4.26	21.52±4.27	22.24±4.22	20.84±4.12	20.31±3.75	20.24±3.52	**0.000**	Level 1–2: 1.84(0.48,3.20)	**0.009**
								Level 1–3: 2.62(1.28,3.97)	**0.000**
Positive Acceptance	17.43±2.85	17.60±2.66	17.67±2.52	17.27±2.39	16.73±2.67	16.58±2.20	0.078	Level 1–2: 0.70(-0.10,1.50)	0.087
								Level 1–3: 0.93(0.06,1.79)	**0.035**
Control for Purpose	9.43±1.88	10.00±2.04	10.07±2.04	9.42±1.75	9.24±1.88	9.13±1.56	**0.024**	Level 1–2: 0.75(0.03,1.47)	**0.042**
								Level 1–3: 0.93(0.18,1.68)	**0.016**
Spiritual Influences	6.48±1.67	6.81±1.37	6.79±1.37	6.09±1.04	6.04±1.15	6.16±1.17	0.236	Level 1–2: 0.38(-0.09,0.85)	0.114
								Level 1–3: 0.24(-0.22,0.71)	0.304
CDRS(total)	79.52±12.81	82.69±13.18	84.19±12.50	78.96±11.51	77.51±12.14	76.71±10.58	**0.000**	Level 1–2: 4.61(1.13,8.09)	**0.010**
								Level 1–3: 6.91(3.47,10.35)	**0.000**

p: p-value, SRI: Stress Response Inventory, CSI: Coping Strategy Indicator, EIQ: Emotional Intelligence Questionnaire, CDRS: Connor-Davidson Resilience Scale, Level 1: baseline, Level 2: 4 weeks, Level 3: 8 weeks, CI: Confidence Interval

**Fig 3:**
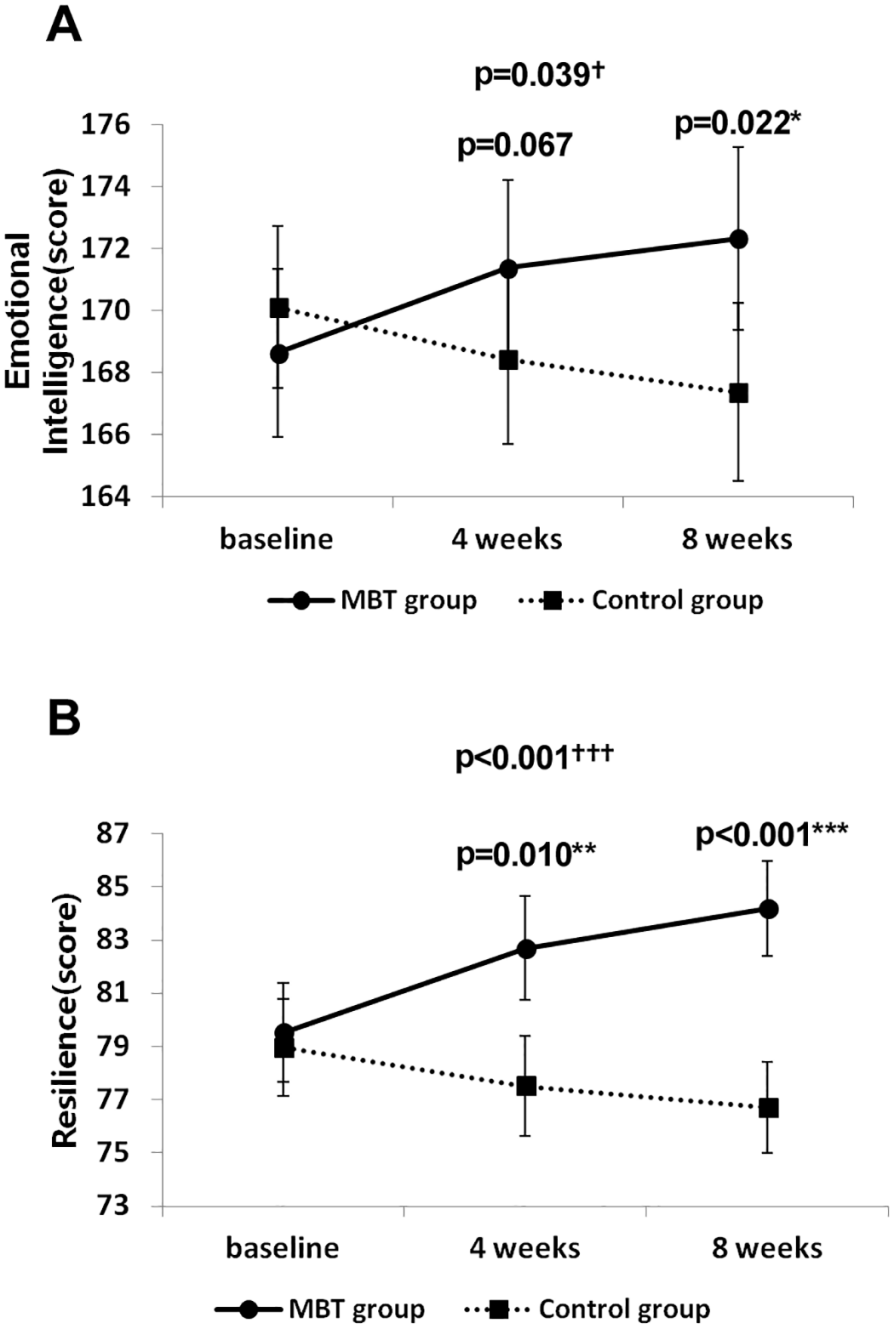
Change in emotional intelligence (A) and resilience (B) scores for the MBT and control groups across the 8 weeks. (A) Significant changes for 8 weeks in emotional intelligence (total score of 5 factors). (B) Significant changes for 8 weeks in resilience (total score of 5 factors). †: Tests of within-subjects effects (time x group). *: Tests of within-subjects of contrasts (time x group).

A significant time x group interaction was found with regard to emotional intelligence (Fig 3A, p = 0.039), with contrast tests indicating a significant interaction effect when comparing baseline vs. 8 weeks (p = 0.022), but not for baseline vs. 4 weeks (Fig 3A), demonstrating an improvement over time in emotional intelligence among MBT participants compared with the control group. Significant time x group interactions were found in emotional thinking (p = 0.036) and emotion regulation (p = 0.033); the results of contrast tests are presented in Table 2. A significant time x group interaction was found with regard to resilience (Fig 3B, p < 0.001), with contrast tests showing a significant interaction effect for baseline vs. 4 weeks (p = 0.010) and baseline vs. 8 weeks (p < 0.001), indicating an enhancement in resilience for the MBT group compared with the control group (Fig 3B). The detailed rmANOVA results for various factors related to resilience are shown in Table 2.

### Effects of MBT on anger and positive and negative affect

Significant time x group interactions were found with regard to state anger (Table 3, p = 0.009) and trait anger/angry temperament (p = 0.013), demonstrating a reduction in anger among MBT participants compared with the control group. A significant time x group interaction was found with regard to negative affect (Table 3, p = 0.042), showing a slight reduction in negative affect among the MBT subjects compared with controls (Table 3). The contrast tests showed a significant time x group interaction with regard to negative affect for baseline vs. 8 weeks (p = 0.009), but not for baseline vs. 4 weeks (Table 3). According to the rmANOVA, there were no significant differences between 4 and 8 weeks with regard to any of contrast tests pertaining to any of the psychological instruments.

**Table 3:** Mean STAXI and PANAS scores for the MBT and control groups at each time point.

	MBT group(M±SD)			Control group(M±SD)			rmANOVA		
	Baseline	4-week	8-week	Baseline	4-week	8-week	Time x group (p)	Group difference in mean change from two levels (95% CI)	p
STAXI									
State Anger	12.24±2.92	11.57±2.72	11.14±2.18	11.38±2.44	12.44±3.79	12.38±3.08	**0.009**	Level 1–2: -1.73(-3.26,-0.21)	**0.026**
								Level 1–3: -2.10(-3.29,-0.90)	**0.001**
Trait Anger	20.12±5.09	19.33±4.86	18.81±4.87	19.31±4.81	19.58±5.53	19.38±4.94	0.193	Level 1–2: -1.05(-2.63,0.52)	0.187
								Level 1–3: -1.38(-2.91,0.15)	0.077
Trait Anger/Angry Temperament	7.67±2.20	7.29±2.36	7.19±2.21	7.11±2.52	7.73±2.81	7.56±2.62	**0.013**	Level 1–2: -1.00(-1.76,-0.24)	**0.010**
								Level 1–3: -0.92(-1.63,-0.21)	**0.012**
Trait Anger/Angry Reaction	9.79±3.10	9.17±2.50	9.07±3.01	9.47±2.32	9.16±2.62	8.91±2.08	0.822	Level 1–2: -0.31(-1.29,0.67)	0.533
								Level 1–3: -0.16(-1.14,0.83)	0.750
PANAS									
Positive Affect	24.90±5.81	28.29±5.50	27.14±6.33	25.98±6.16	27.22±6.04	27.04±6.08	0.264	Level 1–2: 2.14(-0.29,4.56)	0.083
								Level 1–3: 1.17(-1.63,3.98)	0.409
Negative Affect	21.76±6.04	21.76±7.53	20.12±6.39	21.49±6.20	23.87±6.88	23.47±6.70	**0.042**	Level 1–2: -2.38(-5.26,0.51)	0.105
								Level 1–3: -3.62(-6.37,-0.87)	**0.009**

p: p-value, STAXI: State-Trait Anger expression Inventory, PANAS: Positive Affect and Negative Affect Schedule, Level 1: baseline, Level 2: 4 weeks, Level 3: 8 weeks, CI: Confidence Interval

### Associations of perceived stress (SRI) with job demand stress at baseline and in 8-week variations

**Fig 4:**
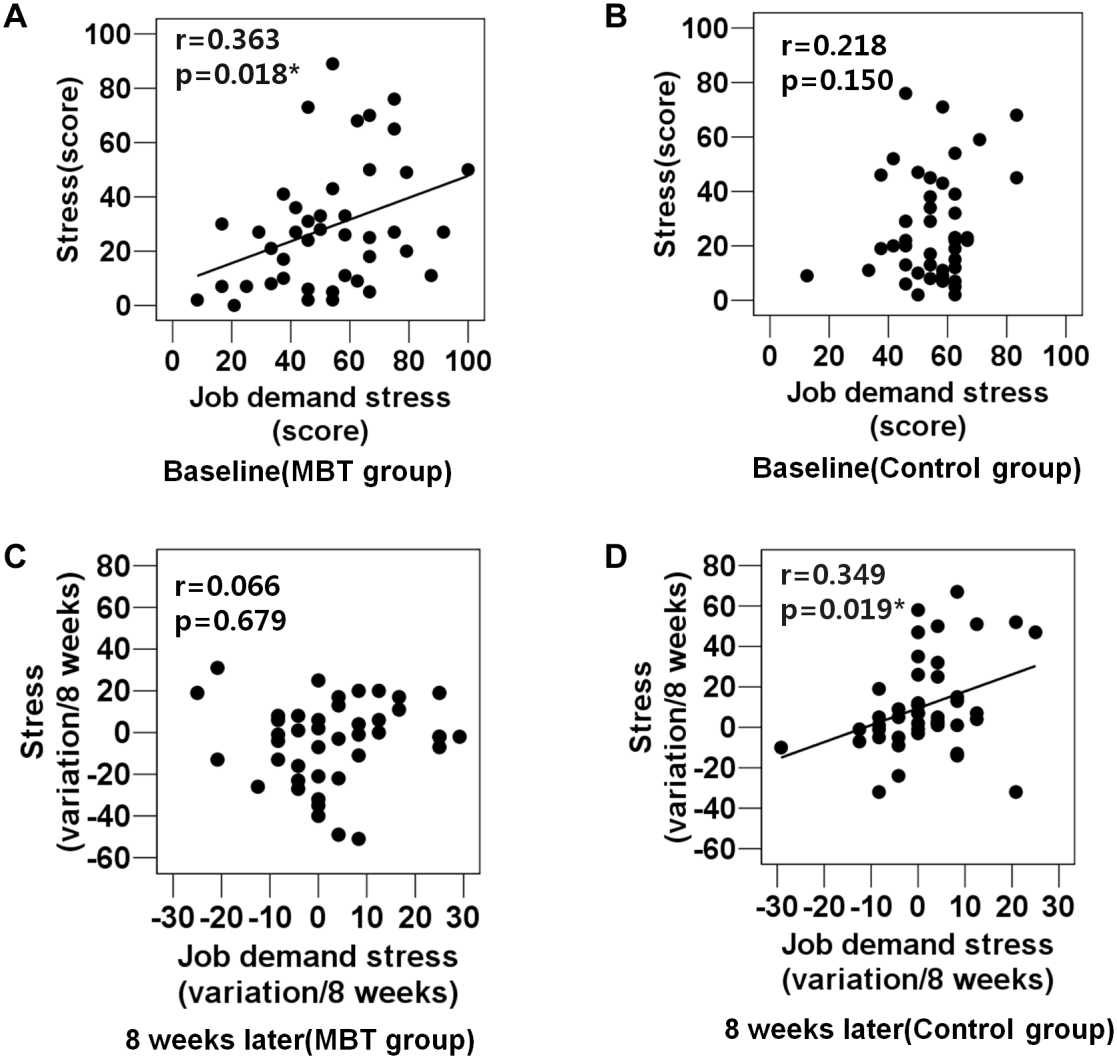
Correlations between job demand stress and perceived stress (SRI) in each group at baseline and in 8-week variations (scores subtracted baseline scores from 8-week scores). (A) A correlation between job demand stress and perceived stress (SRI) in the MBT group at baseline. (B) A correlation between job demand stress and perceived stress (SRI) in the control group at baseline. (C) A correlation between job demand stress (8-week variations) and perceived stress (8-week variations) in the MBT group. (D) A correlation between job demand stress (8-week variations) and perceived stress (8-week variations) in the control group.

**Fig 5:**
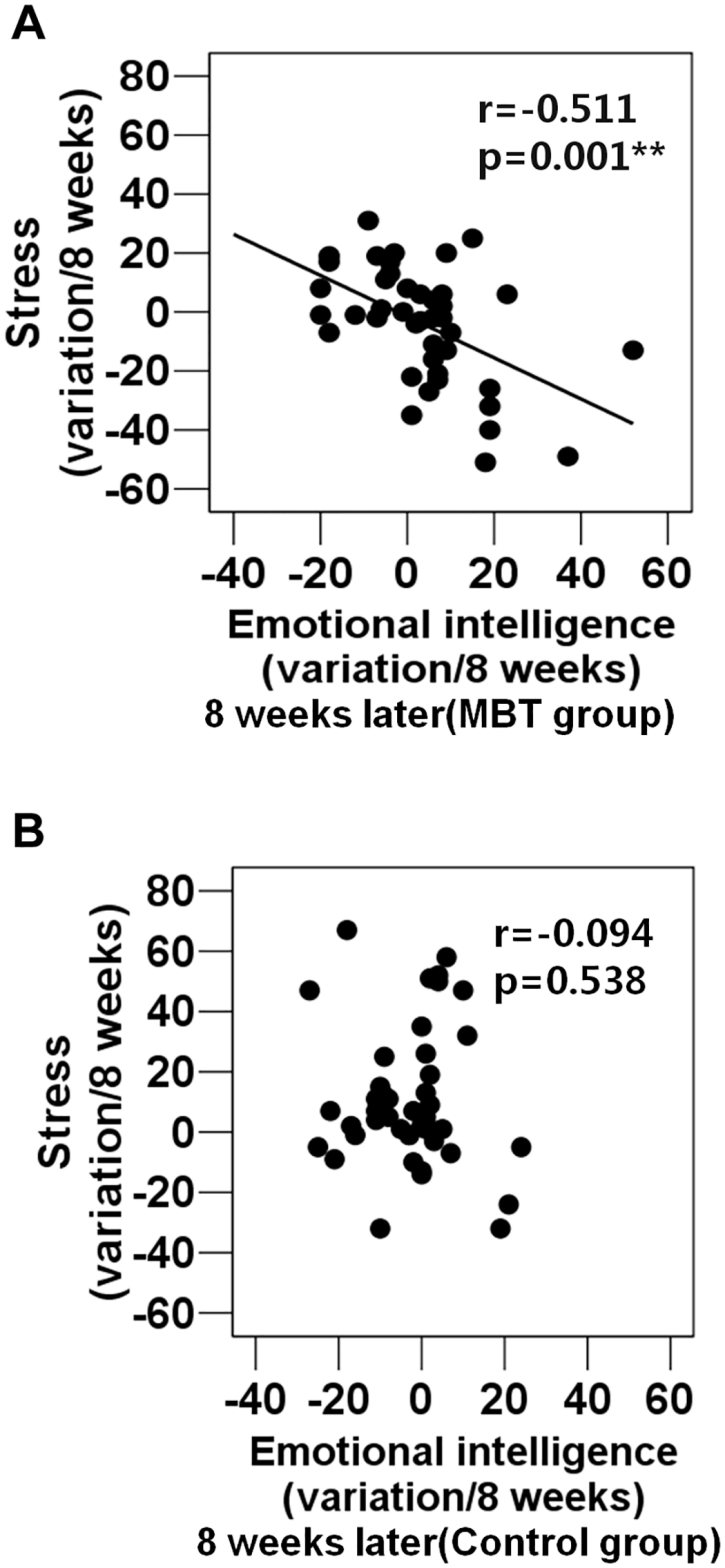
Correlations between emotional intelligence (8-week variations) and perceived stress (8-week variations) in each group. (A) A correlation between emotional intelligence (8-week variations) and perceived stress (8-week variations) in the MBT group. (B) A correlation between emotional intelligence and perceived stress (8-week variations) in the control group.

We analyzed the correlation between targeted variables, focusing on differences in the degree of correlation between the MBT and control groups. At baseline, the two groups exhibited different patterns of correlations (Fig 4A and 4B): the MBT group showed a significant positive correlation between the job demand factor of occupational stress and perceived stress (SRI), as shown (Fig 4A); however, this correlation was not found in the control group at baseline (Fig 4B). On the other hand, the reverse pattern was found at 8 weeks (Fig 4C and 4D): the 8-week variations in job demand stress were significantly correlated with the 8-week variations in perceived stress (SRI) in the control group (Fig 4D) but not in the MBT group (Fig 4C). 8-week variations indicated changed scores which were calculated by subtracting baseline scores from 8-week scores). Positive scores in 8-week variations mean increased scores but negative scores indicate decreased scores (Figs 4C, 4D and 5).

### Associations between stress and emotional intelligence in 8-week variations

Further analysis showed that in 8-week variations, emotional intelligence was negatively correlated with stress (SRI) in the MBT group (Fig 5A); however, this correlation was not observed in the control group (Fig 5B).

## Discussion

The 8-week online MBT program evaluated in this study resulted in significant effects with regard to stress reduction, improvement in the use of effective coping strategies, increased emotional intelligence, enhanced resilience, and decreased anger and negative affect. The reduction in stress associated with MBT can be understood in terms of the decrease in tension, frustration, anger, depression, and somatization during the 8 weeks. As the program consists of training for both mind and body, the stress-related physical and emotional symptoms, such as tension, frustration, anger, depression, and somatization, of participants seemed to improve compared with those of the control subjects.

These effects of MBT seem to be related to improvements in psychological capabilities. Given that subjects in both groups were chosen based on exclusion criteria stipulating an absence of a mental disorder (SCL-90-R), their psychological levels of functioning were within the normal range. Nevertheless, we possess the capacity to improve our psychological well-being, and the MBT program examined in this study seemed to enhance the psychological capabilities and well-being of the participants. Moreover, we found individual differences in the effects of MBT. For example, the stress scores of 24 subjects (57.1%) in the MBT group decreased, and the emotional intelligence scores of 26 subjects (61.9%) in this group increased. Additionally, although the stress scores of the MBT group decreased by an average of four points after 8 weeks, the stress scores of 24 subjects (57.1%) in this group decreased by an the average of 17 points. Likewise, the emotional intelligence scores of 26 subjects (61.9%) in the MBT group increased by an average of 12 points, compared with an average increase of four points in all MBT subjects. Thus, despite individual differences, the effects of the MBT program examined in this study were positive. The causes of such individual differences regarding the effects of MBT should be explored in future research.

We should examine why the control group showed significantly increased stress after 4 and 8 weeks. Although there were no occupation-related differences between the control and experimental groups, we can speculate that workplace stress increased during this time as a result of the outbreak of Middle East Respiratory Syndrome (MERS). Indeed, most subjects in both groups were nurses involved in emotional labor and caring staff, who worked in the hospital. As a result of the MERS emergency, the number of patients visiting doctors decreased during the period of baseline testing. About 1 month later, at the time of the second testing, the MERS emergency had dissipated, and more patients visited the hospital. Indeed, we can speculate that most members of both groups were very busy at work more than 1 month before, but our data reflected no significant differences in occupational stress (e.g., job demands) between the control and experimental groups. Thus, we can infer that a small increase in job-demand stress may have contributed to an increase in the perceived stress of some subjects in the control group (Fig 4D). On the other hand, given that, unlike the control group, the MBT group showed no association between job-demand stress and perceived stress (Fig 4C), it is possible that some subjects in the MBT group improved their stress resistance, which is ability to withstand stress without causing maladjustments or disorders, compared with their baseline results (Fig 4A). These findings are meaningful in that the effects of MBT on stress reduction, along with the other various psychological improvements, were observed despite the fact that participants remained in stressful work situations that showed no significant reduction in total occupational stress over the 8-week period. In other words, subjects who participated in the MBT program appeared to become more resistant to the psychological stress induced by occupational pressures such as job demands than they were before taking part in the program, as demonstrated by the results of the correlation analysis (Fig 4). We can infer from these results that, prior to the 8-week program, the MBT group was vulnerable to stress based on job demands; however, after the 8-week program, the stress level experienced by these participants was not affected by increases in the stress related to job demands. In contrast, at 8 weeks, the control group, who did not receive the intervention, experienced an increase in stress in accordance with increased job demands.

As emotions are related to respiration [43], deep breathing during MBT may affect not only physical but also emotional relaxation. Additionally, meditation for the purpose of emotional release may be beneficial for those engaged in emotional labor. Emotional intelligence, including emotional perception and expression, empathy, and emotion regulation, may be important for those engaged in emotional labor as they are required to display certain emotions in the presence of customers or others [9]. As integrative mind and body training improves attention and self-regulation through the interaction between the central (brain) and the autonomic (body) nervous systems [26], the reduction in stress and the increase in emotional intelligence associated with MBT may contribute to the ability of those involved in professions that require high levels of emotional labor to self-regulate. A crucial factor for the stress reduction observed among the MBT subjects may be the increase in emotional intelligence that resulted from participating in the 8-week program; this is suggested by the inverse correlation between emotional intelligence scores and self-reported stress (Fig 5). We can infer that the increase in emotional intelligence and the reduction in stress may closely and synergistically interact and that such dynamics may be induced or increased by MBT. Thus, this result offers important clues about the hidden mechanisms underpinning the ability of MBT to improve psychological well-being. Previous studies reporting an inverse relationship between stress and emotional intelligence [14, 17] further support our results. Given that emotional intelligence acts as a moderator between stress levels and psychological health [18] and increased emotional competence contributes to the use of adaptive coping strategies when dealing with stress [19], the enhanced emotional intelligence observed among subjects taking part in the MBT program may have led to improved problem-solving and social-support coping as well as a reduction in stress. Although this 8-week online program did not include educational lecture programs specifically targeting coping strategies, subjects who participated in the program showed an increase in the frequency with which they used adaptive coping strategies. The healthy brain has a considerable capacity for resilience based on its ability to respond to interventions designed to open "windows of plasticity" and redirect its function toward better health [44]. Thus, given that meditation may potentially strengthen neuronal circuits and enhance cognitive reserve capacity [45], the MBT program may contribute to the brain capacity and plasticity of healthy subjects, leading to more frequent use of adaptive coping strategies and reductions in stress.

Moreover, adaptive coping strategies such as problem-solving and social-support strategies along with increased emotional intelligence appeared to increase stress resistance in our subjects. The improved resilience achieved through MBT, reflected in our results, is similar to findings of previous studies showing immediate improvements in resilience after online mind–body skills training [8] and the long-term effects of mindfulness training on resilience [46], in that MBT contributed to enhanced resilience. As resilience is associated with an individual's ability to appropriately adapt to stress and adversity [21], the improvement in resilience through MBT seems to be related to the reduction in stress and the increased use of adaptive coping strategies.

Our participants also exhibited a decrease in the negative effect commonly associated with stress. It has been found that severe stress induces anger [47]; the decrease in anger and negative affect we observed among our participants in the 8-week program seems to be associated with the reduction of stress they exhibited. Furthermore, lower negative affect was associated with greater emotional intelligence [48]. Our own results further suggest that an increase in emotional intelligence, an increased use of adaptive coping strategies and a greater resilience among subjects participating in the 8-week training program seem to be associated with reduced stress, anger, and negative affect.

The effects of this online MBT program on psychological states were similar to those of the previous offline MBT, which included the same techniques. For example, like the previous offline MBT [22], the online MBT program was associated with decreased stress. The previous offline MBT was associated with an increase in positive affect [22], but this online MBT was correlated with a decrease in negative affect but not an increase in positive affect. As subjects who participated in this 8-week MBT program underwent a much shorter training period than subjects in the previous offline MBT study who had been practicing meditation for a mean of 43 months, some differences between the two studies are to be expected. However, as this online MBT program was shown to decrease stress, increase adaptive coping strategies, increase emotional intelligence, and enhance resilience, additional research comparing the online with the offline approach is needed. Additionally, other online training programs and interventions designed to teach mind–body skills have been reported to affect stress, mindfulness, resilience, empathy, anxiety, and depression [7–8,49]. Thus, we should develop and study effective online training and intervention programs for mind and body.

The majority of the effects we observed of the online MBT program on various psychological measures reached statistical significance at the 4-week mark, but a few factors did not reach significance until 8 weeks. This indicates that although some significant benefits of the online MBT program were apparent at 4 weeks, 8 weeks were needed to achieve broader and more robust effects.

Our results are consistent with previously observed effects of coping, stress reduction and resilience programs on stress, depression, anxiety, anger, coping skills and mindfulness [6, 50] and the effects of online mind–body skills training on stress, resilience, mindfulness, empathy, and compassion [7–8]. Furthermore, given that beneficial effects of mindfulness and resilience training on stress, anger, anxiety, and depression have been observed among patient populations [51–52], the 8-week program investigated in the current study has potential applications for the development of treatment programs for patients.

Although we used a longitudinal design, this study has several limitations. First, we did not use randomized controlled trials. Nevertheless, considering that subjects who participated in the 8-week online program were volunteers whose baseline levels of stress and emotional intelligence were not significantly different from those of participants in the control group, we can be fairly confident in our interpretation of the effects of this program on various psychological factors. A second potential limitation is that the subjects who participated in this study were all female. However, meditation training programs have been found to improve depressive symptoms regardless of religious affiliation, gender or age [2], with little in the way of observed gender differences in previous studies of programs and their effects.

In conclusion, the results of this study suggest that an online MBT program may be beneficial in fostering diverse psychological capabilities including the enhancement of stress resistance, the use of adaptive coping strategies, emotional intelligence, and resilience, and in reducing anger and negative affect.

## Supporting Information

S1 Appendix Available raw data. (SAV)
